# Current concept of abdominal sepsis: WSES position paper

**DOI:** 10.1186/1749-7922-9-22

**Published:** 2014-03-27

**Authors:** Massimo Sartelli, Fausto Catena, Salomone Di Saverio, Luca Ansaloni, Mark Malangoni, Ernest E Moore, Frederick A Moore, Rao Ivatury, Raul Coimbra, Ari Leppaniemi, Walter Biffl, Yoram Kluger, Gustavo P Fraga, Carlos A Ordonez, Sanjay Marwah, Igor Gerych, Jae Gil Lee, Cristian Tranà, Federico Coccolini, Francesco Corradetti, James Kirkby-Bott

**Affiliations:** 1 Department of Surgery, Macerata Hospital, Macerata, Italy; 2 Department of Emergency Surgery, Maggiore Parma Hospital, Parma, Italy; 3 Trauma Surgery Unit, Maggiore Hospital, Bologna, Italy; 4 General Surgery Department, Papa Giovanni XXIII hospital, Bergamo, Italy; 5 American Board of Surgery, Philadelphia, USA; 6 Department of Surgery, Denver Health Medical Center, Denver, CO, USA; 7 Department of Surgery, University of Florida, Gainesville, Florida, USA; 8 Department of Surgery, Virginia Commonwealth University Medical Center, Richmond, VA, USA; 9 Department of Surgery, UC San Diego Health System, San Diego, USA; 10 Department of Abdominal Surgery, University Hospital Meilahti, Helsinki, Finland; 11 Department of General Surgery, Rambam Health Care Campus, Haifa, Israel; 12 Division of Trauma Surgery, Hospital de Clinicas -, School of Medical Sciences, University of Campinas, Campinas, Brazil; 13 Department of Surgery, Universidad del Valle, Fundacion Valle del Lili, Cali, Colombia; 14 Department of Surgery, Pt BDS Post-graduate Institute of Medical Sciences, Rohtak, India; 15 Department of Surgery 1, Lviv Regional Hospital, Danylo Halytsky Lviv National Medical University, Lviv, Ukraine; 16 Department of Surgery, Yonsei University College of Medicine, Seoul, Korea; 17 Department of Anesthesiology, Macerata Hospital, Macerata, Italy; 18 Department of Surgery, University Hospital Southampton, Southampton, UK

## Abstract

Although sepsis is a systemic process, the pathophysiological cascade of events may vary from region to region.

Abdominal sepsis represents the host’s systemic inflammatory response to bacterial peritonitis.

It is associated with significant morbidity and mortality rates, and is the second most common cause of sepsis-related mortality in the intensive care unit.

The review focuses on sepsis in the specific setting of severe peritonitis.

## Introduction

Abdominal sepsis is associated with significant morbidity and mortality rates.

Results of prospective trials have often overestimated the outcomes of patients with severe peritonitis [1]. Treatment of patients who have complicated intra-abdominal infections (IAIs) by adequate management, has generally been described to produce satisfactory results; recent clinical trials have demonstrated an overall mortality of 2% to 3% among patients with complicated IAIs [1,2].

However, results from published clinical trials may not be representative of the true morbidity and mortality rates of such infections. Patients who have perforated appendicitis are usually over represented in clinical trials [1]. Furthermore patients with intra-abdominal infection enrolled in clinical trials have often an increased likelihood of cure and survival. In fact trial eligibility criteria often restrict the inclusion of patients with co-morbid diseases that would increase the death rate of patients with intra-abdominal infections.

After excluding patients with perforated appendicitis, Merlino et al. [3] found that the cure rate among patients who had intra-abdominal infections and were enrolled in clinical trials, was much higher than that of patients who were not enrolled (79% versus 41%) and that the mortality rate was much lower (10% versus 33%).

Epidemiological studies of patients with intra-abdominal infections including severely ill subjects, have demonstrated higher mortality rates [4].

In the CIAO study the overall mortality rate was 7.7% (166/2152) [5]. Analyzing the subgroup of patients with severe sepsis or septic shock at admission to hospital the mortality rate reached 32.4% (89/274). In patients with severe sepsis or septic shock in the immediate post-operative period, the mortality rate was 42.3% (110/266).

Abdominal sepsis represents the host’s systemic inflammatory response to bacterial or yeast peritonitis.

In the event of peritonitis gram-negative, gram-positive, as well as anaerobic bacteria, including common gut flora, such as *Escherichia coli, Klebsiella pneumoniae*, *Streptococcus spp.* and *Bacteroides fragilis*, enter the peritoneal cavity. Sepsis from an abdominal origin is initiated by the outer membrane component of gram-negative organisms (e.g., lipopolysaccharide [LPS], lipid A, endotoxin) or gram-positive organisms (e.g., lipoteichoic acid, peptidoglycan), as well anaerobe toxins. This lead to the release of proinflammatory cytokines such as tumor necrosis factor α (TNF-α), and interleukins 1 and 6 (IL-1, IL-6). TNF-α and interleukins lead to the production of toxic mediators, including prostaglandins, leukotrienes, platelet-activating factor, and phospholipase A2, that damage the endothelial lining, leading to increased capillary leakage [6]. Cytokines lead to the production of adhesion molecules on endothelial cells and neutrophils. Neutrophil-endothelial cell interaction leads to further endothelial injury through the release of neutrophil components. Activated neutrophils release nitric oxide, a potent vasodilator that leads to septic shock. Cytokines also disrupt natural modulators of coagulation and inflammation, activated protein C (APC) and antithrombin. As a result, multiple organ failure may occur.

Early detection and timely therapeutic intervention can improve the prognosis and overall clinical outcome of septic patients. However, early diagnosis of sepsis can be difficult; determining which patients presenting with signs of infection during an initial evaluation, do currently have, or will later develop a more serious illness is not an easy or straightforward task.

Sepsis is a complex, multifactorial syndrome which can evolve into conditions of varying severity. If left untreated, it may lead to the functional impairment of one or more vital organs or systems [7].

Severity of illness and the inherent mortality risk escalate from sepsis, through severe sepsis and septic shock up multi-organ failure.

Previous studies have demonstrated that mortality rates increase dramatically in the event of severe sepsis and septic shock [8]. Severe sepsis may be a reasonable approximation of the “tipping point” between stable and critical clinical conditions in the management of intra-abdominal infections. Severe sepsis is defined as sepsis associated with at least one acute organ dysfunction, hypoperfusion, or hypotension.

It is well known that hypotension is associated with an increased risk of sudden and unexpected death in patients admitted to hospital with non traumatic diseases [9]; identifying patients with severe sepsis early and correcting the underlying microvascular dysfunction may improve patient outcomes. If not corrected, microvascular dysfunction can lead to global tissue hypoxia, direct tissue damage, and ultimately, organ failure [10].

The Surviving Sepsis Campaign international guidelines for management of severe sepsis and septic shock were recently updated [11]. These guidelines are the cornerstone for the management of severe sepsis and septic shock, but they do not focus on the specific setting of intra-abdominal infections.

Although sepsis is a systemic process, the pathophysiological cascade may vary from organ to organ.

There are few data regarding systemic and local responses during peritonitis in humans and on their correlation to patients outcomes [12-14].

Based on findings of high concentrations of cytokines in the peritoneal compartment, some evidences suggested that intra-abdominal sepsis may result in a cytokine-mediated inflammatory response that is initially compartmentalized in the peritoneal cavity [15,16].

Animal models have shown that peritonitis is associated with a significant and prolonged peritoneal inflammatory response which is adversely correlated with survival outcome [17].

The levels of selected peritoneal cytokines have been reported to be significantly different between animals that survived as compared to those who died following a septic challenge [18].

Plausibility of peritoneal compartmentalization of initial inflammatory response during peritonitis was highlighted by a recent prospective cohort study of patients with secondary generalized peritonitis [19]. It confirmed that IL-1, TNFα, IL-6, IL-10 and IFNγ are present at high concentrations in the peritoneal fluid of patients with peritonitis. The results of this study showed a large gradient between peritoneal fluid and plasma concentrations of cytokines, with no correlation between peritoneal and plasma levels, suggesting that plasma levels may increase only after saturation of tissues within the abdominal compartment.

The inflammatory response in patients with sepsis depends on the causative pathogen and the host (genetic characteristics and coexisting illnesses), with differential responses at local, regional, and systemic levels [20].

The host inflammatory response probably changes over time in parallel with the clinical course. Sepsis, in the early stages of the inflammatory process, should be considered as a local/peritoneal disease. In advanced stages, severe sepsis and septic shock should be considered as a systemic disease, and patients who are extremely unstable and exhibit high rates of mortality should be managed more aggressively.

In certain patients peritonitis can quickly lead to an excessive inflammatory response, and early and aggressive mechanical peritoneal control is determinant for stopping the septic process. In those patients inability to control or interrupt the local inflammatory response is associated with poor outcomes.

In patients with ongoing sepsis, several laparotomies may be required. Under these circumstances, open abdomen allows the surgeon to perform subsequent laparotomies more efficiently and prevent the onset of abdominal compartment syndrome that may further worsen the systemic disease.

The review focuses on management of patients with severe sepsis or septic shock in the specific setting of severe peritonitis.

## Diagnosis

Reducing time to diagnosis of severe sepsis is thought to be a critical component in reducing mortality from sepsis-related multiple organ dysfunction [11]. Delineating the source of infection as accurately as possible prior to surgery is the primary aim and the first step in managing intra-abdominal infections. In severe abdominal sepsis however, delays in operative management may lead to worse outcomes and early exploration is always recommended when peritonitis is suspected even if the source of infection is not recognized pre-operatively with certainty.

The diagnosis of intra-abdominal sepsis is based primarily on clinical assessment. Typically, the patient is admitted to the emergency department with abdominal pain and a systemic inflammatory response, including fever, tachycardia, and tachypnoea. Abdominal rigidity suggests the presence of peritonitis. However, clinical assessment alone is not always reliable in critically ill patients due to a variety of clinical constraints (e.g., impaired consciousness, severe underlying disease, etc.). Hypotension, oliguria, and acute altered mental status are waring signs of the patient’s transition from sepsis to severe sepsis.

Plain abdominal films are often the first imaging obtained for patients presenting with peritonitis. Upright films are useful for identifying free air under the diaphragm (most often on the right side), which can result from perforated viscera. Free air may be present in most cases of anterior gastric and duodenal perforation. However it is much less frequent with perforations of the small bowel and colon and is unusual with appendiceal perforation. Abdominal plain films have low sensitivity and specificity, and have, in most cases, been replaced by abdominal computed tomography (CT). However, plain films of the abdomen remain a reasonable initial study for patients with suspected peritonitis who, on the basis of history and physical examination, are likely candidates for surgical exploration. In this case, abdominal plain films may confirm evidence of perforation in short time.

Ultrasonography and computed tomography have become essential diagnostic tools in abdominal sepsis. The diagnostic approach to confirm the source of abdominal infection in septic patients depends largely on the haemodynamic stability of the patient [21].

Critically ill patients who are haemodynamically unstable or have developed severe acute respiratory distress syndrome (ARDS) requiring high-level ventilatory support, are at significant risk during transport to the radiology department In unstable patients who do not undergo an immediate laparotomy and whose critical condition prevents them from leaving ICU for further imaging, ultrasound (US) is the best available imaging modality [22]. It is portable, it can be performed at the bed side, it is reproducible and can be easily repeated. Major drawbacks are ileus and obesity, which may significantly mask the US view. US is also strongly operator-dependent. In suspected biliary sepsis US is always the preferred initial diagnostic modality for acute cholecystitis and emphysematous cholecystitis.

In stable patients, abdominal computerized tomography (CT) is the imaging modality of choice, especially when the diagnosis is uncertain. However, in patients with severe sepsis, if the diagnosis of peritonitis is made clinically or by previous radiological examinations (plain films of the abdomen or US), additional CT scanning may be unnecessary and would only delay much-needed surgical intervention [22].

Another option in the diagnosis of critically ill patients suffering from intra-abdominal sepsis is bedside laparoscopy, as it can avoid patient transport to the radiological department or operating room is very accurate, and maintains ICU monitoring [23]. Laparoscopy provides a “minimally invasive” definitive modality to diagnose intra-abdominal sepsis. It may quickly provide the necessary information to address further management. However, the overall mortality of patients undergoing diagnostic laparoscopy in the ICU is high, regardless of diagnostic findings during this procedure. The use of diagnostic laparoscopy should be limited to patients in whom a therapeutic intervention is strongly suspected [24].

## Antimicrobial therapy

A key component of the first-line management of the septic patient is the administration of IV antimicrobial therapy. Antimicrobial therapy plays a pivotal role in the management of intra-abdominal infections, especially in patients with severe sepsis who require immediate empiric antibiotic therapy.

An insufficient or otherwise inadequate antimicrobial regimen is one of the variables more strongly associated with unfavorable outcomes in critical ill patients [25]. Empiric antimicrobial therapy should be started as soon as possible in patients with severe sepsis with or without septic shock [26-28].

A prospective observational study by Riché et al. involving 180 patients with secondary generalized peritonitis, reported significantly higher mortality rates in patients presenting with septic shock (35%) compared to those presenting without it (8%) [29].

The role of the infecting pathogen on the patients response in secondary peritonitis has been poorly investigated.

Some authors support the concept of a ‘generic septic response’ in which an identical immune response is triggered by any type of bacteria [30,31].

Contrastingly, others suggest that different types of pathogens may elicit various inflammatory responses, despite a common pathway of activation.

Riche et al. have found that polymicrobial cultures or anaerobes in the peritoneal fluid were associated with more frequent septic shock [29].

A recent prospective cohort study showed that patients in whom anaerobes or *Enterococcus* species [19] were isolated from peritoneal fluid cultures released more TNFα in their plasma than those who were infected with other strains. The hypothesis that different types of pathogens may elicit various inflammatory responses, was already highlighted in animal models. In rats with peritonitis, Montravers *et al*. showed that adjunction of *Enterococcus faecalis* was associated with increased mortality as well as higher levels of TNFα and IL-6 in peritoneal fluid [32,33].

Evidence regarding a specific role of some pathogens on the pattern of the sepsis response is rather small, preventing any definitive conclusion from these results.

However it is well known that patients with severe sepsis or septic shock may benefit from aggressive antimicrobial treatment in order to curb the spread of the multiple organ dysfunction syndrome caused by an ongoing peritoneal trigger.

For these patients, a de-escalated approach may be the most appropriate strategy. Increasing rates of resistance and a more comprehensive understanding of the sepsis process have prompted many experts to advocate the use of broad-spectrum antimicrobial regimens in the initial stages of treatment for sepsis [34,35]. Subsequent modification (de-escalation) of the initial regimen becomes possible later, when culture results are available and clinical status can be better assessed, 48–72 hours after initiation of empiric therapy.

When treating abdominal sepsis, clinicians must be aware that drug pharmacokinetics may differ significantly between patients due to the variable pathophysiology of sepsis, and must also take into account the pathophysiological and immunological status of the patient [36].

The “dilution effect”, also called the ‘third spacing’ phenomenon, must be considered when administering hydrophilic agents such as β-lactams, aminoglycosides, and glycopeptides, which selectively distribute to the extracellular space. Low plasma antimicrobial levels can contribute to lower than expected antimicrobial concentrations in peritoneal fluid with potentially reduced antimicrobial delivery to the target tissues. In fact, the target plasma concentration (Ct) that should be achieved with the loading dose (LD) depends solely on the volume of distribution (Vd) of the drug (LD = Ct × Vd). If the Vd is enlarged the Ct will results in a lower than expected level with the standard LD [36].

Higher than standard loading doses of β-lactams, aminoglycosides, or glycopeptides should be administered to ensure optimal drug exposure to the infection site in patients with severe sepsis or septic shock [36].

Lastly it should be kept in mind that the loading dose of lipophilic antibiotics (Macrolides, Fluoroquinolones, Tetracyclines, Chloramphenicol, Rifampicin, Linezolid) which are not influenced by the “diluition effect”, should not be influenced by the severe sepsis or septic shock status [36].

Once appropriate initial loading is achieved, it is mandatory to reassess the antimicrobial regimen daily, because the pathophysiological changes that may occur, may significantly affect drug disposition in the critically ill patients. Lower than standard dosages of renally excreted drugs must be administered in the presence of impaired renal function, while higher than standard dosages of renally excreted drugs may be needed for optimal exposure in patients with glomerular hyperfiltration [36].

In Table 1 recommended dosing regimens of the most frequently used renally excreted antimicrobials according to renal function are illustrated.

**Table 1:** **Recommended dosing regimens of the most frequently used renally excreted antimicrobials according to renal function**[21]

	**Renal function**			
**Antibiotic**	**Increased**	**Normal**	**Moderately impaired**	**Severely impaired**
Piperacillin/tazobatam	16/2 g q24 h CI or 3.375 q6 h EI over 4 hours	4/0.5 g q6 h	3/0.375 g q6 h	2/0.25 g q6 h
Imipenem	500 mg q4 h or 250 mg q3 h over 3 hours CI	500 mg q6 h	250 mg q6 h	250 mg q12 h
Meropenem	1 g q6 h over 6 hours CI	500 mg q6 h	250 mg q6 h	250 mg q12 h
Ertapenem	ND	1 g q24 h	1 g q24 h	500 mg q24 h
Gentamycin	9 to 10 mg/kg q24 h^b^	7 mg/kg q24 h	7 mg/kg q36–48 h	7 mg/kg q48–96 h
Amikacin	20 mg/kg q24 h	15 mg/kg q24 h	15 mg/kg q36–48 h^b^	15 mg/kg q48–96 h
Ciprofloxacin	600 mg q12 h or 400 mg q8 h	400 mg q12 h	400 mg q12 h	400 mg q24 h
Levofloxacin	500 mg q12 h	750 mg q24 h	500 mg q24 h	500 mg q48 h
Vancomycin	30 mg/kg q24 h CI	500 mg q6 h	500 mg q12 h	500 mg q24–72 h
Teicoplanin	LD 12 mg/kg q12 h for 3 to 4 doses; MD 6 mg/kg q12 h	LD 12 mg/kg q12 h for 3 to 4 doses; MD 4 to 6 mg/kg q12 h	LD 12 mg/kg q12 h for 3 to 4 doses; MD 2 to 4 mg/kg q12 h	LD 12 mg/kg q12 h for 3 to 4 doses; MD 2 to 4 mg/kg q24 h
Tigecycline	LD 100 mg; MD 50 mg q12 h	LD 100 mg; MD 50 mg q12 h	LD 100 mg; MD 50 mg q12 h	LD 100 mg; MD 50 mg q12 h

Regarding the administration of antibiotics, treatment efficacy against a certain microorganism can involve the specific drug concentration and/or the time when the drug is introduced to the binding site [36].

Concentration-dependent antibiotics, such as aminoglycosides and quinolones, are more effective at higher concentrations. They therefore feature a concentration-dependent post-antibiotic effect, and bactericidal action continues for a period of time after the antibiotic level falls below the minimum inhibitory concentration (MIC) [36].

Concentration-dependent agents administered in high dosage, short-course, once-a-day treatment regimens may promote more rapid and efficient bactericidal action and prevent the development of resistant strains.

There is good evidence for extended duration of aminoglycoside dosing in critically ill patients. In terms of toxicity, aminoglycosides nephrotoxicity is caused by a direct effect on the renal cortex and the uptake into the renal cortex can be saturated. Thus a dosing strategy of extended duration reduces the renal cortex exposure to aminoglycosides and reduces the risk of nephrotoxicity [37].

Time-dependent antibiotics, such as β-lactams and glycopeptides, demonstrate optimal bactericidal activity when drug concentrations are maintained above the MIC. Unlike concentration-dependent agents, they have a negligible post-antibiotic effect.

The efficacy of time-dependent antibacterial agents in severely ill patients is based on the constant maintenance of supra-inhibitory drug concentrations; as such, clinicians should consider multiple doses per day [38].

In critically ill patients, continuous infusion of β-lactam antibiotics may facilitate faster and more consistent therapeutic levels as compared to intermittent bolus dosing. Although randomized clinical trials are needed to confirm these findings, continuous infusion of β-lactam antibiotics has proven to be a useful time-dependent approach for treating critically ill patients [39].

The empirically designed antimicrobial regimen is based on the underlying severity of infection, the pathogens presumed to be involved, and the risk factors indicative of major resistance patterns.

Intra-abdominal infections in critically ill patients can be treated with either single or multiple antimicrobial regimens depending on the range requirements of antimicrobial coverage [40].

Piperacillin/tazobactam is a beta-lactam/beta-lactamase inhibitor combination with in vitro activity towards gram-positive (including Enterococci), gram-negative and anaerobic organisms [41].

Piperacillin/tazobactam retains in vitro activity against broad-spectrum beta-lactamase-producing, many extended-spectrum beta-lactamase-producing Enterobacteriaceae and many *Pseudomonas* isolates [42]. It is still a good antimicrobial agent in critically ill patients with community-acquired intra-abdominal infections.

Carbapenems have a spectrum of antimicrobial activity that includes Gram-positive (except resistant gram positive cocci) and Gram-negative aerobic and anaerobic pathogens.

Group 2 carbapenems include imipenem/cilastatin, meropenem and doripenem, sharing activity against non-fermentative gram-negative bacilli and being particularly suitable for severe intra-abdominal infections [43].

Doripenem is a new 1-ß-methyl carbapenem which, similarly to imipenem and meropenem, has a broad-spectrum activity against Gram-positive, Gram-negative, and anaerobic bacteria [44]. Doripenem seems more effective, in vitro, than meropenem and imipenem against Pseudomonas aeruginosa [44].

In the last few years carbapenem overuse has been associated with increasing rates of resistance among enterobacteriacea [45], particularly *Klebsiella pneumonia.* From an epidemiological point of view, it is necessary to control the spread of carbapenemase producing gram negative bacteria by optimization of carbepenems use. The use of carbapenems in critically ill patients is acceptable and well indicated. Tigecycline represents a valid option for complicated intra-abdominal infections due to its favorable in vitro activity against enterococci, ESBL-producing strains of *E. coli* and *Klebsiella* and anaerobic organisms. Tigecycline has showed also considerable antimicrobial activity against *Acinetobacter* spp [46,47]. It does not have in vitro activity towards *Pseudomonas aeruginosa* and *Proteus mirabilis.*

Given its in vitro activity against multidrug resistant (MDR) bacteria, tigecycline represents an interesting treatment option for intra-abdominal infections at risk for MDR [48].

Recently, an analysis of clinical trials for both approved and unapproved indications for tigecycline (including one trial on complicated intra-abdominal infections), showed an increased risk of death among patients receiving tigecycline. This observation led to a FDA recommendation against the use of tigecycline in severe infections [49].

Because of its tissue penetration in peritoneal and soft tissues [50], tigecycline is a very useful drug used in peritoneal infections. In patients with severe sepsis or septic shock of abdominal origin, in which the inflammatory process extends to the circulatory system, tigecycline should always be associated with another antimicrobial.

Although the epidemiological role of candida species in intra-abdominal infections has not yet been conclusively defined by the medical community, the clinical role of candida is nevertheless significant given that invasive candidiasis is generally associated with poor clinical prognosis. However, the presence of *Candida* in patients with no signs of infection is considered a contaminant and may not require treatment.

Fluconazole has been widely used for the treatment of candidiasis since its approval by the FDA in 1990.

The azoles act primarily by inhibiting the cytochrome P450-dependent enzyme lanosterol 14-alpha-demethylase, necessary for the conversion of lanosterol to ergosterol in the cellular membrane of fungi [51].

Most *C. albicans* isolated from invasive candidiasis infections, remain fully susceptible to fluconazole, which has been the treatment of choice for these infections in most settings including intra-abdominal infections [52]. However, epidemiological data demonstrate that the frequency of *Candida* infections is rising, with an increase in the proportion of infections caused by non-albicans *Candida* species that are intrinsically resistant or variably susceptible to fluconazole [52].

Several randomized clinical trials have demonstrated the efficacy of the echinocandins in the treatment of candidaemia and invasive candidiasis [53].

The echinocandins: anidulafungin, caspofungin, and micafungin have a broad and similar spectrum of in vitro and in vivo activity against most Candida spp. [54].

Echinocandins have several potential advantages over fluconazole for the treatment of invasive candidiasis. They have a broader spectrum of activity (encompassing fluconazole-resistant *C. glabrata* and *C. krusei*) and potent fungicidal activity against most Candida species [55].

In the specific setting of intra-abdominal infections, echinocandins are generally recommended as a first line empiric therapy for critical ill patients, while fluconazole is typically recommended for less severe cases [21].

## Haemodynamic support

One of the most likely explanations for the high morbidity and mortality rates associated with severe sepsis is the development of cardiovascular insufficiency, which can lead to global tissue hypoxia.

In severe sepsis, the early haemodynamic profile is characterized by hypovolaemia, vaso-regulatory dysfunction, and myocardial depression. Increased capillary leakage and venous capacitance ultimately result in decreased venous return to the heart. Additionally, cytokines released during the patient’s immune response may trigger further myocardial depression.

These haemodynamic alterations associated with the early stages of sepsis are often accompanied by an increase in systemic oxygen demand and impaired oxygen delivery, thereby inducing global tissue hypoxia. Global tissue hypoxia may overstimulate endothelial cell activity, which can subsequently lead to the systemic inflammatory cascade characteristic of sepsis [56,57].

Early treatment with aggressive haemodynamic support can limit the damage of sepsis-induced tissue hypoxia and prevent the over stimulation of endothelial activity.

Rivers et al. [58] demonstrated that early goal-directed therapy (EGDT), initiated in the emergency department, reduces the in-hospital mortality rates of patients in septic shock.

It has been established that the general prognostic value of a lactate of 4 mM/L on hospital admission is important; multiple studies have confirmed the risk stratification of this lactate level for illness severity and mortality in both the pre-hospital and in-hospital setting [59-63]. Lactate clearance has also been associated with decreased mortality in patients with severe sepsis and septic shock [64]. However, 20 to 50% of septic shock patients do not have elevated lactate levels at presentation or during their clinical course, yet still develop organ failure [65-67].

### Fluid resuscitation

Fluid resuscitation should be initiated as early as possible in the course of treatment for severe sepsis regardless of a patient’s lactate level.

Fluid resuscitation is a major component of cardiovascular support in early sepsis. Although the need for fluid resuscitation in sepsis is well established, the goals and components of this treatment are still a matter of debate also in patients with peritonitis.

The absence of clear benefits following the administration of colloid solutions compared to crystalloid [68], supports a high-grade recommendation for the use of crystalloid solutions in the initial resuscitation of patients with severe sepsis and septic shock [11].

Intravascular volume is the first parameter to be assessed during hemodynamic optimization.

In patients with generalized peritonitis, fluid resuscitation should be kept under control to avoid fluids overload, which may aggravate gut oedema and lead to increased intra-abdominal pressure. Increasing intra-abdominal pressure causes progressive hypoperfusion of splanchnic circulation. Pathophysiological effects include gut oedema leading to bacterial translocation and release of cytokines, therefore aggravating the sepsis cascade [69].

Several studies have already shown that a positive fluid balance in critical illness may be strongly associated with a higher severity of organ dysfunction and with worse outcomes [70].

Pathophysiological mechanisms associated with the inflammatory response lead to capillary leakage. Although crystalloids are isotonic, a significant amount of the volume given may migrate into the extra-vascular space due to increased capillary permeability and changes in oncotic pressure.

In patient with severe generalized peritonitis excessive infusion of fluids may become a counterproductive strategy.

The frequency with which intra-abdominal hypertension develops in abdominal sepsis may have other important clinical consequences in addition to its impact on sepsis resuscitation endpoints. Current surviving sepsis guidelines emphasize the importance of traditional mean arterial pressure (MAP) >65 mm Hg, central venous pressure (CVP) of 8–12 mmHg in combination with a central venous oxygen saturation (ScvO2) > 70% and Urine output >0.5 mL/kg/hr [11]. However, in patients with severe sepsis or septic shock of abdominal origin, high intra-abdominal pressure may profoundly influence commonly used septic shock resuscitation endpoints such as CVP (falsely elevated) and urine output (markedly decreased).

Repeated intravesical measurements of intra-abdominal pressure should be frequently performed in patients with severe sepsis or septic shock of abdominal origin, to identify patients at risk for intra-abdominal hypertension.

Monitoring the fluid status of critically ill patients at risk for intra-abdominal hypertension is crucial. In recent decades we have witnessed rapid advances in fluid monitoring techniques. Pulmonary artery catheters (PACs) have been widely used for more than three decades, but their usefulness in improving patient outcomes seems disappointing. Trials have consistently shown that PACs do no improve patient outcomes and may significantly increase medical costs [71]. With the declining use of PACs, there has been an increasing number of alternatives for hemodynamic monitoring.

Echocardiography is a useful noninvasive tool which can directly visualize the heart and assess cardiac function. Its use was long limited by the absence of accurate indices to diagnose hypovolemia and predict the effect of volume expansion. In the last years echocardiography has been used to develop new parameters of fluid responsiveness, taking advantage of its ability to monitor cardiac function. Echocardiography has been shown to predict fluid responsiveness accurately and is now a complete and noninvasive tool able to accurately determine hemodynamic status in circulatory failure [72,73]. It is strongly operator-dependent, and it does not allow continuous monitoring.

The PiCCO system (Pulse index Contour Continuous Cardiac Output, Pulsion Medical Systems, Germany) is another interesting alternative. It incorporates a transpulmonary thermodilution technique (TPTD) and continuous pulse contour analysis. It is minimally invasive and does not require intracardiac catheterization. It can give beat-by-beat monitoring of cardiac output, and can provide accurate information on volume status [74].

### Vasopressor agents

Vasopressor agents should be administered early in patients with severe sepsis or septic shock of abdominal origin to restore organ perfusion. Their early use may prevent excessive fluid resuscitation.

Vasopressor drugs maintain adequate blood pressure and preserve perfusion pressure thus optimizing blood flow in various organs. Norepinephrine is now the first-line vasopressor agent used to correct hypotension in the event of septic shock [11]. Norepinephrine is more efficacious than dopamine and may be more effective for reversing hypotension in patients with septic shock.

In 1993, Martin et al. showed in a prospective, double-blind, randomized trial that norepinephrine was more effective and reliable than dopamine to reverse the abnormalities of hyper dynamic septic shock [75]. The Surviving Sepsis Campaign guidelines favour norepinephrine [11] and there have been studies since the 2008 update to bolster this preference. De Backer et al. investigated this question in a meta-analysis, focusing only on those patients with septic shock and again showed that dopamine was associated with greater mortality than norepinephrine [76].

It is well known that dopamine may cause more tachycardia and may be more arrhythmogenic than norepinephrine [77], and as an alternative vasopressor agent to norepinephrine, it should be used only in patients with low risk of tachyarrhythmias and absolute or relative bradycardia.

Epinephrine is a potent α-adrenergic and β-adrenergic agent that increases mean arterial pressure by increasing both, cardiac index and peripheral vascular tone. There are concerns regarding the use of epinephrine in septic patients due to its potential to decrease regional blood flow, particularly in the splanchnic circulation, and elevations in serum lactate. However, no trials have shown that epinephrine results in worse outcomes, so it may be used as an alternative to norepinephrine [78,79].

Vasopressin is a peptide hormone synthesized in the hypothalamus and subsequently transported to the pituitary gland where it is stored. It is released in response to decreased blood volume, decreased intravascular volume, and increased plasma osmolality. Vasopressin constricts vascular smooth muscle by directly activating V1 receptors and simultaneously increasing the vasculature’s responsiveness to catecholamines [80].

Vasopressin (up to 0.03 U/min) can be added to norepinephrine with the intent of raising MAP to target or decreasing the norepinephrine dose [11].

### Inotropic agents

Dobutamine is frequently used to treat septic shock patients as an inotropic agent increasing cardiac output, stroke index, and oxygen delivery (Do_2_). However, the tendency of dobutamine to increase Do_2_ to supra-normal values in critically ill patients has raised serious questions regarding its safety in the treatment of septic shock. The Surviving Sepsis Campaign Guidelines recommend [11] that a dobutamine infusion should be administered in the event of myocardial dysfunction as indicated by elevated cardiac filling pressures and low cardiac output or ongoing signs of hypoperfusion, despite achieving adequate intravascular volume and adequate MAP.

### Acute kidney injury in surgical sepsis

In patients with surgical sepsis, particular attention should always be paid to acute kidney injury (AKI). A prospective observational institutional study recently published, has shown that AKI frequently complicates surgical sepsis, and serves as a powerful predictor of hospital mortality in severe sepsis and septic shock. During the 36-month study period ending on December 2010, 246 patients treated for surgical sepsis were evaluated in the study. AKI occurred in 67% of all patients, and 59%, 60%, and 88% of patients had sepsis, surgical sepsis, and septic shock, respectively.

Patients with AKI had fewer ventilator-free and intensive care unit free days and a decreased likelihood of discharge to home. Morbidity and mortality increased with severity of AKI, and AKI of any severity was found to be a strong predictor of hospital mortality (odds ratio, 10.59; 95% confidence interval, 1.28Y87.35; p = 0.03) in surgical sepsis [81].

## Source control

### Initial operation

The timing and adequacy of source control are of outmost importance in the management of intra-abdominal sepsis, as late and/or incomplete procedures may have severely adverse consequences on outcome.

Source control encompasses all measures undertaken to eliminate the source of infection, reduce the bacterial inoculum and correct or control anatomic derangements to restore normal physiologic function [82,83].

This generally involves drainage of abscesses or infected fluid collections, debridement of necrotic or infected tissues and definitive control of the source of contamination.

It is well known that inadequate source control at the time of the initial operation has been associated with increased mortality in patients with severe intra-abdominal infections [84].

Early control of the septic source can be achieved using both operative and non-operative techniques.

An operative intervention remains the most viable therapeutic strategy for managing intra-abdominal sepsis in critical ill patients.

The initial aim of the surgical treatment of peritonitis is the elimination of bacterial contamination and inflammatory substances and prevention or reduction, if possible, of fibrin formation.

Generally, the surgical source control employed depends on the anatomical source of infection, the degree of peritoneal inflammation and generalized septic response, and the patient’s pre-morbid condition.

Surgical source control entails resection or suture of a diseased or perforated viscus (e.g. diverticular perforation, gastroduodenal perforation), removal of the infected organ (e.g. appendix, gallbladder), debridement of necrotic tissue, resection of ischemic bowel and repair/resection of traumatic perforations with primary anastomosis or exteriorization of the bowel.

Laparotomies are usually performed using a midline incision.

The primary objectives of surgical intervention include a) determining the cause of peritonitis, b) draining fluid collections, c) controlling the origin of the abdominal sepsis.

Special attention should be given to areas where abscesses may form such as the pelvis, the para-colic gutters, and the subphrenic spaces. These areas should be carefully exposed and debrided, avoiding bleeding by excessive peeling of the fibrin, and drained.

In case of suspected gastro-intestinal perforation, the whole extent of the GI tract, starting from the gastroesophgeal junction to the lower rectum should be thoroughly and carefully examined. If no perforation is found, the gastrocolic omentum should always be opened to expose the lesser sac to allow visualization of the posterior wall of stomach for any hidden perforation as well as careful examination of the body and tail of pancreas.

Special attention should be paid while draining and debriding the left subphrenic space since there is high risk of splenic injury during surgical manipulation due to fibrinous adhesions with the splenic capsule. Splenic bleeding maybe difficult to control due to adhesions and might warrant splenectomy which adds to the morbidity and potential mortality in an already compromised patient.

Intra-abdominal lavage is a matter of ongoing controversy. Some authors have favoured peritoneal lavage because it helps in removal as well as in dilution of peritoneal contamination by irrigation with great volumes of saline [85]. However, its application with or without antibiotics in abdominal sepsis is largely unsubstantiated in the literature [86].

In recent years, laparoscopy has been gaining wider acceptance in the diagnosis and treatment of intra-abdominal infections.

Laparoscopic approach in the treatment of peritonitis is feasible and effective without any specific complications in experienced hands. Laparoscopy has the advantage to allow, at the same time, an adequate diagnosis and appropriate treatment with the less invasive abdominal approach [87]. However, in unstable patients laparoscopy is generally avoided because increased intra-abdominal pressure due to pneumoperitoneum seems to have a negative effect in critical ill patients leading to acid–base balance disturbances, as well as changes in cardiovascular and pulmonary physiology [88].

### Relaparotomy strategy

In certain circumstances, infection not completely controlled may trigger an excessive immune response and sepsis may progressively evolve into severe sepsis, septic shock, and organ failure [89].

Such patients would benefit from immediate and aggressive surgical treatment with subsequent re-laparotomy strategies, to curb the spread of organ dysfunctions caused by ongoing sepsis.

Unfortunately, early assessment of the severity of peritonitis is difficult in emergency surgical patients and none of the existing and widely used ‘severity-of-disease’ scores, specifically developed for critically ill patients, were clinically useful in the identification of patients with ongoing infection needing a re-laparotomy [90]. Surgical strategies following an initial emergency laparotomy include subsequent “re-laparotomy on demand” (when required by the patient’s clinical condition) as well as planned re-laparotomy in the 36-48-hour post-operative period.

On-demand laparotomy should be performed only when absolutely necessary and only for those patients who would clearly benefit from additional surgery.

Several studies have evaluated clinical variables that may be associated with the need for on-demand re-laparotomy in the immediate post-operative period [91-97].

Van Ruler et al. [92] in 2008 reported the results of a questionnaire asking surgeons to rank the importance of 21 clinical variables on their decision to re-operate in patients with secondary peritonitis. They found that diffuse extent of the abdominal contamination, localization of the infectious focus (upper gastrointestinal tract including small bowel), and both, extremely low and high leukocyte counts, independently predicted a re-laparotomy. These variables had only moderate predictive accuracy. The results of the questionnaire demonstrated that there was no consensus among surgeons about which variables are important in the decision-making process for re-laparotomy. The final decision to perform a re-operation on a patient in the on-demand setting is generally based on the patients generalized septic response and on the lack of clinical improvement.

Performing a case–control study, Koperna and Schulz [91] retrospectively reviewed 523 consecutive patients with secondary peritonitis. They focused their attention on 105 patients, in whom standard surgical treatment of secondary peritonitis failed and who had to undergo re-laparotomy for persisting abdominal sepsis (study group). The authors showed that patients re-operated on after 48 hours had a significantly higher mortality rate than those operated on earlier (76.5% versus 28%; p < .001).

Planned relaparotomies, on the other hand, are performed every 36–48 hours for purposes of inspection, drainage, and peritoneal lavage of the abdominal cavity.

The concept of a planned relaparotomy for severe peritonitis has been debated for over thirty years. Re-operations are performed every 48 hours for reassessing the peritoneal inflammary process until the abdomen is free of ongoing peritonitis; then the abdomen is closed. The advantages of the planned re-laparotomy approach are optimization of resource utilization and reduction of the potential risk for gastrointestinal fistulas and delayed hernias.

The results of a clinical trial published in 2007 investigating the differences between on-demand and planned re-laparotomy strategies in patients with severe peritonitis found few advantages for the planned re-laparotomy strategy; however, the study mentioned that this later group exhibited a reduced need for additional re-laparotomies, decreased patient dependency on subsequent health care services, and decreased overall medical costs [98].

### Open abdomen

An open abdomen (OA) procedure is the best way of implementing re-laparotomies. The role of the OA in the management of severe peritonitis has been a controversial issue.

In 2007, a randomised study compared open and closed abdomens for the “on demand re-laparotomy” group in the treatment of severe peritonitis. The study was prematurely terminated following the treatment of 40 subjects due to a significantly higher mortality rate in the open abdomen group compared to the temporarily closed abdomen group (55% vs. 30%). OA procedures were performed using only non-absorbable polypropylene mesh [99].

Although guidelines suggest not to routinely utilize the open abdomen approach for patients with severe intra-peritoneal contamination undergoing emergency laparotomy for intra-abdominal sepsis [100], OA has now been accepted as a strategy in treating intra-abdominal sepsis [101].

An OA approach in severe secondary peritonitis may be required for three different reasons, often used in combination: inadequate source control, severely deranged physiology (the operation is purposely abbreviated due to the severe physiological derangement and suboptimal local conditions for healing, and restoration of intestinal continuity is deferred to the second operation, i.e. the deferred anastomosis approach) [102], and prevention of abdominal compartment syndrome [103-105].

The rationale of the OA strategy in patients with severe abdominal sepsis refers to the cytokine release that is compartmentalized in the peritoneal cavity. Inability to control or interrupt the local inflammatory response is associated with higher mortality rates in these patients. The attenuation of the local inflammatory response may be best achieved with mechanical control by reducing the load of cytokines and other inflammatory substances [106] and by preventing their production, thus removing the source itself. Sometimes more laparotomies are required to complete source control and OA allows the surgeon to perform subsequent planned laparotomies more efficiently.

An interesting non-comparative descriptive case series [106] studied the inflammatory response in peritoneal exudate and plasma of patients undergoing planned re-laparotomy for severe secondary peritonitis. In septic patients undergoing re-laparotomy for severe peritonitis, endotoxin, tumour necrosis factor alpha, interleukin-1 and interleukin-6 levels, were higher in the peritoneal cavity then in plasma. When patients underwent re-laparotomy, the level of those cytokines was significantly decreased in survivors.

OA management has been described in patients with intra-abdominal sepsis when a single laparotomy failed to control local inflammatory response, or the risk of organ dysfunction increased after effective drainage and debridement [107-109].

In the event of massive fluid resuscitation, bowel oedema and the forced closure of a non-compliant abdominal wall may cause intra-abdominal hypertension (IAH). Uncontrolled IAH exceeding 25 mm Hg may cause abdominal compartment syndrome (ACS), which is a potentially lethal complication characterized by adverse effects on pulmonary, cardiovascular, renal, splanchnic, and central nervous system physiology [109].

The combination of IAH and the physiological effects of sepsis, result in high morbidity and mortality rates. At present there are no definite criteria to guide the surgeon in deciding whether to use the OA strategy [110]. The OA strategy allows surgeons to extend the concept of damage control surgery to abdominal severe sepsis.

The term damage control surgery (DCS) for trauma patients was introduced in 1993. It was defined as initial control of haemorrhage and contamination, allowing for resuscitation to normal physiology in the intensive care unit and subsequent definitive re-exploration [111,112].

The adaptation of damage control surgery for trauma to other areas generally is useful in those patients who are at risk to develop a similar loss of physiologic reserve with intolerance to the shocked physiological state [113]. Similarly to the trauma patient with the lethal triad of acidosis, hypothermia and coagulopathy, many patients with severe sepsis or septic shock may present in a similar fashion. For those patients, DCS can truly be life saving. Patients progressing from sepsis through severe sepsis with organ dysfunction into septic shock, can present with vasodilation, hypotension, and myocardial depression, combined with coagulopathy. These patients are profoundly haemodynamically unstable and are clearly not optimal candidates for complex operative interventions [114].

Abdominal closure should be temporary, and the patient is rapidly taken to the ICU for physiologic optimization. This includes optimization of volume resuscitation and mechanical ventilation, correction of coagulopathy and hypothermia, and monitoring for eventual ACS developement. Over the following 24 to 48 hours, when abnormal physiology is corrected the patient can be safely taken back to the operating room for re-operation.

An additional advantage of DCS in abdominal sepsis is the possibility to delay the bowel anastomosis [115].

The surgical strategy for the management of patients with compromised bowel in secondary peritonitis has been usually the resection of the perforated viscus followed by primary anastomosis or a diversion. In patients with severe secondary peritonitis and significant hemodynamic instability and compromised tissue perfusion, the use of primary anastomosis is limited because of the high risk of suture/anastomotic failure, leakage, and increased surgical mortality. In these patients, it is advisable to control the source of peritoneal contamination and to perform an intestinal ostomy delaying bowel anastomosis.

In a retrospective study from Colombia, 112 patients with secondary peritonitis requiring bowel resection and managed with staged laparotomy were analyzed [116]. Deferred primary anastomosis was used in 34 patients where the bowel ends were closed at first operation and definitive anastomoses were reconstructed at the subsequent operation following physiological stabilization in the ICU and repeated peritoneal washes until the septic source was controlled. In contrast, 78 patients underwent small bowel or colonic diversion followed by similar ICU stabilization and peritoneal washes. In both groups, the abdomens were left open at the initial operation and a Velcro system or vacuum pack was used for temporary abdominal closure. The mean number of laparotomies was four in both groups. There were more patients with colon resections in the diversion group (80% vs. 47%). There was no significant difference in hospital mortality (12% for deferred anastomosis vs. 17% for diversion), frequency of anastomotic leaks or fistulas (9% vs. 5%), or ARDS (18% vs. 31%). The authors concluded that in critically ill patients with severe secondary peritonitis managed with staged laparotomies, deferred primary anastomosis can be performed safely as long as adequate control of the septic foci and restoration of deranged physiology is achieved prior to reconstruction.

In a non-randomized study of 27 consecutive patients with perforated diverticulitis (Hinchey III/IV), the patients were managed either with sigmoid resection and primary anastomosis, or limited sigmoid resection or suture, open abdomen and primary anastomosis or colostomy at second operation 24–48 hours later, or Hartmann procedure; sigmoid resection and end colostomy [117]. All 6 patients with primary anastomosis survived without complications, but there was an obvious selection bias. Of the 6 patients undergoing Hartmann’s procedure, one died of sepsis and 5 were discharged with stoma. In the interesting group of 15 patients with deferred anastomosis or stoma and open abdomen, 9 patients had intestinal continuity restored during the second look operation with one fatal anastomotic leakage.

In a prospective study of 51 patients with perforated diverticulitis (Hinchey III/IV) were initially managed with limited resection, lavage and TAC with vacuum-assisted closure followed by second, reconstructive operation 24–48 hours later [118]. Bowel continuity was restored in 38 patients, in 4 protected by a loop ileostomy. Five anastomotic leaks (13%) were encountered requiring loop ileostomy (2 patients) or Hartmann’s procedure (3 patients). Postoperative abscesses were seen in 4 patients, abdominal wall dehiscence in one and re-laparotomy for drain-related small bowel perforation in one. The overall mortality rate was 10% and 35/46 (76%) of the surviving patients left the hospital with reconstructed colon continuity. Fascial closure was achieved in all patients.

Following stabilization of the patient, the goal is the early and definitive closure of the abdomen, in order to reduce the complications associated with an open abdomen [119].

A review of the literature suggests a bimodal distribution of primary closure rates, with early closure dependent on post operative intensive care management whilst delayed closure is more affected by the choice of the temporary abdominal closure technique [120].

Primary fascial closure can be achieved in many cases within few days from the initial operation. It would not be successful if early surgical source control failed [121,122].

Sequential fascial closure could immediately be started once abdominal sepsis is well controlled [123]. In these cases, surgeons should perform a progressive closure, where the abdomen is incrementally closed each time the patient undergoes a reoperation.

Within 10 to 14 days the fascia retracts laterally and becomes adherent to the overlying fat; this makes primary closure impossible. Therefore, it is important to prevent the retraction of the myo-fascial unit.

Several materials can be used to achieve temporary closure of the abdomen: gauze; mesh; impermeable self-adhesive membrane dressings, zippers and negative pressure therapy (NPT) techniques.

The ideal temporary abdominal closure method should be able to protect the abdominal contents, to prevent evisceration, to allow removal of infected or toxic fluid from the peritoneal cavity, to prevent the formation of fistulas, to avoid damage to the fascia, to preserve the abdominal wall domain, to make re-operation easy, safe and facilitate definitive closure [110].

The surgical options for management of the OA are now more diverse and sophisticated, but there is a lack of prospective randomized controlled trials demonstrating the superiority of any particular method.

At present, negative pressure therapy (NPT) techniques have become the most extensively used methods for temporary abdominal wall closure. NPT actively drains toxin or bacteria-rich intra peritoneal fluid and has resulted in a high rate of fascial and abdominal wall closure [110].

A systematic review conducted in 2012 [124] found only 11 comparative studies, including 2 randomized controlled trials (RCTs) and 9 cohort studies, examining the efficacy and safety of negative pressure peritoneal therapy *versus* alternate temporal abdominal closure methods among critically ill or injured adults.

However, all studies were associated with at least a moderate risk of bias and significant clinical heterogeneity, the authors concluded that there was insufficient evidence to support the preferential use of negative pressure peritoneal therapy after damage control laparotomy.

Animal data suggest that OA techniques employing constant negative pressure to the peritoneal cavity may remove inflammatory ascites, reduce the systemic inflammatory response, and improve organ injury and potentially outcomes [125].

This method is still associated with high morbidity and high incidence of ventral hernia formation in surviving patients caused by difficulties in definitive closure of the abdominal wall after prolonged application of NPT but it could be a highly promising method in the management of patients with increased IAP and severe sepsis due to severe peritonitis [126].

A systematic review published in 2009 [127] investigated which temporary abdominal closure technique is associated with the highest delayed primary fascial closure (FC) rate.

No comparative studies were identified. 51 articles were included. The techniques described were vacuum-assisted closure (VAC; 8 series), vacuum pack (15 series), artificial burr (4 series), Mesh/sheet (16 series), zipper (7 series), silo (3 series), skin closure (2 series), dynamic retention sutures (DRS), and loose packing (1 series each).

These results suggested that the artificial burr and the VAC were associated with the highest FC rates and the lowest mortality rates.

Other techniques used for progressive FC include a combination of NPT with a temporary mesh sutured to the fascial edges. The mesh is tightened every few days, until the fascial defect is small enough so the mesh can be removed and the fascia closed primarily.

In 2012, a retrospective analysis evaluating the use of vacuum-assisted closure and mesh-mediated fascial traction (VACM) as temporary abdominal closure was published [128]. The study compared 50 patients treated with (VACM) and 54 using non-traction techniques (control group). VACM resulted in a higher fascial closure rate and lower planned hernia rate than methods that did not provide fascial traction.

Occasionally, abdominal closure is only partially achieved, resulting in late development of large, debilitating hernias of the abdominal wall which will eventually require complex surgical repair. In these cases, delayed repair or use of biological meshes has been proposed [129].

Another option, if definitive fascial closure is not possible, is closure of the skin only and subsequent management of the eventration by a deferred abdominal closure with synthetic meshes after hospital discharge [127].

## Adjuntive measures

Recombinant human activated protein C (rhAPC), also known as drotrecogin alfa, was included in the previous Surviving Sepsis Campaign guidelines [130] based on the PROWESS study group [131] and ENHANCE study group [132] studies.

Based on the preliminary data of the PROWESS-SHOCK study [133], showing a 28-day all-cause mortality rate of 26.4% in patients treated with rhAPC compared with 24.4% in those given placebo, the US Food and Drug Administration (FDA) has withdrawn drotrecogin alfa from the market [134] and now, rhAPC should not be used in any patients with septic shock.

### Corticosteroids

The recommendations regarding the use of corticosteroids have changed over time and the clinical benefits of corticosteroids in the treatment of severe sepsis and septic shock remain controversial.

A systematic review of corticosteroids in the treatment of severe sepsis and septic shock in adult patients published in 2009 valued 17 randomized trials (2138 patients) and 3 quasi-randomized trials (n = 246) of acceptable methodological quality, and pooled the results in a subsequent meta-analysis [135]. The authors concluded that corticosteroid therapy has been used in varied doses for treating sepsis and related syndromes for more than 50 years, but its ability to reduce mortality rates has never been conclusively proven. Since 1998, studies have consistently used prolonged low-dose corticosteroid therapy, and follow-up analyses of this subgroup have found that such regimens tend to reduce short-term mortality.

In 2011 Annane published an evidenced based guide [136] regarding corticosteroids for severe sepsis. He concluded that corticosteroids should be initiated only in patients with sepsis who require 0.5 μg/kg per minute or more of norepinephrine and should be continued for 5 to 7 days except in patients with poor haemodynamic response after 2 days of corticosteroids and with a cortisol increment of more than 250 nmol/L after a standard adrenocorticotropin hormone (ACTH) test.

The Surviving Sepsis Campaign guidelines [11] recommend corticosteroids be used in patients with refractory septic shock (poorly responsive to fluids and vasopressor therapy) and do not recommend routine assessment for relative adrenal insufficiency.

### Nutritional support

The effect of nutritional support in critically ill patients with sepsis has been debated in recent years. As for all critically ill patients, nutritional support, preferably via the enteral route, should be commenced in patients with severe sepsis or septic shock once initial resuscitation and adequate perfusion pressure is achieved [137].

Early enteral nutrition has theoretical advantages in maintaining the integrity of the gut mucosa and on the prevention of bacterial translocation.

Studies on different subpopulations of critically ill patients, mostly surgical patients, are not consistent and none was individually powered for mortality, with very low mortality rates. Although no consistent effect on mortality was observed, some early enteral feeding studies showed benefit on secondary outcomes such reduced length of mechanical ventilation, and reduced ICU and hospital stay [138-140].

## Conclusions

The Surviving Sepsis Campaign international guidelines for management of severe sepsis and septic shock were recently updated. These guidelines are the cornerstone for the management of severe sepsis and septic shock, but they do not focus on the specific setting of intra-abdominal infections.

Although sepsis is a systemic process, the pathophysiological events differ for every organ and in the specific setting of intra-abdominal infections the management of sepsis may vary from that of sepsis of other etiologies.

Outcomes of severe intra-abdominal infections accompanied by severe sepsis are related to early diagnosis, aggressive and early optimization of physiology, early surgical management with source control and aggressive critical care management. Reoperations are common and may be useful in attenuating the inflammatory response and optimizing the immune response.

## Competing interests

The authors declare that they have no competing interests.

## Authors’ contributions

MS wrote the manuscript. All authors reviewed and approved the final manuscript.
